# Supplementary material for: HIVprotI: an integrated web based platform for prediction and design of HIV proteins inhibitors
Source: J Cheminform. 2018 Mar 9;10:12. doi: 10.1186/s13321-018-0266-y (PMC5845081; doi:10.1186/s13321-018-0266-y)
Supplement: Supplementary file 2 — Additional file 2. Source code of HIVProtI web server. [file 13321_2018_266_MOESM2_ESM.zip › HIVprotI_Source-code/hivprotI/weka-3-6-6/doc/weka/core/pmml/PMMLModel.html]

PMMLModel


---


|  |  |  |
| --- | --- | --- |
| **Overview** \| **Package** \| **Class** \| **Tree** \| **Deprecated** \| **Index** \| **Help** \| **Weka's home** | |  |
| **PREV CLASS**   **NEXT CLASS** | **FRAMES**    **NO FRAMES**     **All Classes** |  |
| SUMMARY: NESTED \| FIELD \| CONSTR \| METHOD | DETAIL: FIELD \| CONSTR \| METHOD |  |


---


## weka.core.pmml Interface PMMLModel

**All Known Implementing Classes:**: GeneralRegression, NeuralNetwork, PMMLClassifier, Regression

---

``` public interface PMMLModel ```

Interface for all PMML models

**Version:**
:   $Revision: 5562 $

**Author:**
:   Mark Hall (mhall{[at]}pentaho{[dot]}com)

---

| **Method Summary** | |
| --- | --- |
| `java.lang.String` | `getCreatorApplication()`             Get the name of the application that created this model. |
| `Logger` | `getLog()`             Get the logger. |
| `MiningSchema` | `getMiningSchema()`             Get the mining schema. |
| `java.lang.String` | `getPMMLVersion()`             Get the version of PMML used to encode this model. |
| `void` | `setCreatorApplication(org.w3c.dom.Document doc)`             Set the name of the application (if specified) that created this. |
| `void` | `setLog(Logger log)`             Set a logger to use. |
| `void` | `setPMMLVersion(org.w3c.dom.Document doc)`             Set the version of the PMML. |

**Method Detail**

### setPMMLVersion

```
void setPMMLVersion(org.w3c.dom.Document doc)
```

:   Set the version of the PMML.

    :   **Parameters:**: `doc` - the Document encapsulating the pmml

---


### getPMMLVersion

```
java.lang.String getPMMLVersion()
```

:   Get the version of PMML used to encode this model.

    :   **Returns:**: the version as a String

---


### setCreatorApplication

```
void setCreatorApplication(org.w3c.dom.Document doc)
```

:   Set the name of the application (if specified) that created this.
    model

    :   **Parameters:**: `doc` - the Document encapsulating the pmml

---


### getCreatorApplication

```
java.lang.String getCreatorApplication()
```

:   Get the name of the application that created this model.

    :   **Returns:**: the name of the creating application or null if not specified in the pmml.

---


### getMiningSchema

```
MiningSchema getMiningSchema()
```

:   Get the mining schema.

    :   **Returns:**: the mining schema

---


### setLog

```
void setLog(Logger log)
```

:   Set a logger to use.

    :   **Parameters:**: `log` - the logger to use

---


### getLog

```
Logger getLog()
```

:   Get the logger.

    :   **Returns:**: the logger (or null if none is being used)


---


|  |  |  |
| --- | --- | --- |
| **Overview** \| **Package** \| **Class** \| **Tree** \| **Deprecated** \| **Index** \| **Help** \| **Weka's home** | |  |
| **PREV CLASS**   **NEXT CLASS** | **FRAMES**    **NO FRAMES**     **All Classes** |  |
| SUMMARY: NESTED \| FIELD \| CONSTR \| METHOD | DETAIL: FIELD \| CONSTR \| METHOD |  |


---
